# Anti-MDA5 Positive Dermatomyositis Associated with Rapidly Progressive Interstitial Lung Disease and Correlation between Serum Ferritin Level and Treatment Response

**DOI:** 10.31138/mjr.31.1.75

**Published:** 2020-03-31

**Authors:** Daniela Noa Zohar, Lior Seluk, Hagith Yonath, Yehuda Shoenfeld, Shaye Kivity

**Affiliations:** 1 Department of Neurology, The Chaim Sheba Medical Center, Ramat Gan, Israel,; 2 Department of Internal Medicine A, The Chaim Sheba Medical Center, Ramat Gan, Israel,; 3 The Zabludowicz Center for Autoimmune Diseases, Sheba Medical Center, Tel Hashomer, Sackler Faculty of Medicine, Tel-Aviv University, Tel Aviv Israel

**Keywords:** Anti-MDA5, clinically amyopathic dermatomyositis, interstitial lung disease, MSA, hyperferritinemia

## Abstract

Clinically amyopathic dermatomyositis is an uncommon autoimmune disorder in the Middle East. The clinical picture of clinically amyopathic dermatomyositis is characterized mainly by pulmonary and dermatological manifestations. Occasionally muscle symptoms are observed as well. Serum anti-MDA5 autoantibody positivity is associated with rapidly progressive interstitial lung disease among clinically amyopathic dermatomyositis patients. Moreover, high serum ferritin level is correlated with poor prognosis and high mortality. Herein we describe the case of an Israeli patient with rapidly progressive interstitial lung disease and without pathognomonic dermatological features who was diagnosed with anti-MDA5 positive clinically amyopathic dermatomyositis and did not survive despite immunomodulatory therapy followed by reduction in serum ferritin levels.

## INTRODUCTION

Clinically amyopathic dermatomyositis is a heterogenous autoimmune disorder involving skin, muscle and internal organs. Although no objective clinical evidence of muscle weakness is observed, some clue for myopathy can be detected in subclinical examinations.^1,2^ Myositis specific auto-antibodies allude about unique clinical features and complications of the disease.^1 ,2,4, 5^ Anti-melanoma differentiation associated gene 5 (anti-MDA5) predicts the development of an interstitial lung disease, principally, rapidly progressive interstitial lung disease that is correlated with hyperferritinemia in dermatomyositis patients.^1,3,5^

## CASE HISTORY

A previously healthy 69-year-old Israeli was admitted to the medical ward for evaluation of a progressive general weakness accompanied by facial rash, productive cough, arthralgia, dysphagia, oral ulcers and a weight loss of approximately 7kg for the past three months. Initial physical examination revealed a “heliotrope rash”, painful oral ulcers and bibasilar crackles on lung auscultation. No objective muscle weakness was recorded. Laboratory findings showed high CPK (884IU/l) and hyperferritinemia (2401ng/ml). Myositis-specific autoantibodies panel was positive for anti-MDA5 autoantibody.

**Figure 1.**
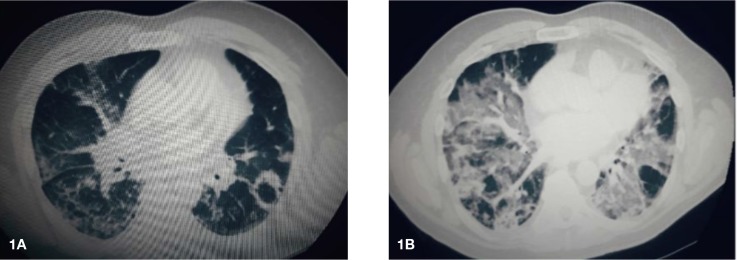
Chest Computed Tomography: A. Third day of hospitalization- bilateral circular reversed halo infiltrates, ground glass opacities mainly in right lower lobe. B. Day 39th since the first admission, CT showed significant progression of diffuse lung disease with many Ground glass opacities.

During the hospitalization, his respiratory state deteriorated and oxygen support was required. Chest CT demonstrated bilateral ground glass opacities on lung bases (*Figure 1A*), pulmonary function tests showed a moderate-severe restrictive pattern. Bronchoalveolar lavage ruled out malignancy and bacterial infection. Nonspecific mild inflammatory myopathy was seen in the deltoid muscle biopsy and characteristic features of dermatomyositis were observed in the cutaneous biopsy. The diagnosis of anti-MDA5 positive clinically amyopathic dermatomyositis with rapidly progressive interstitial lung disease was formulated. The patient received two cycles of methylprednisolone therapy and one course of intravenous cyclophosphamide (750mg/d) with a clinical improvement and was discharged with an oral prednisone treatment and oxygen support.

**Figure 2.**
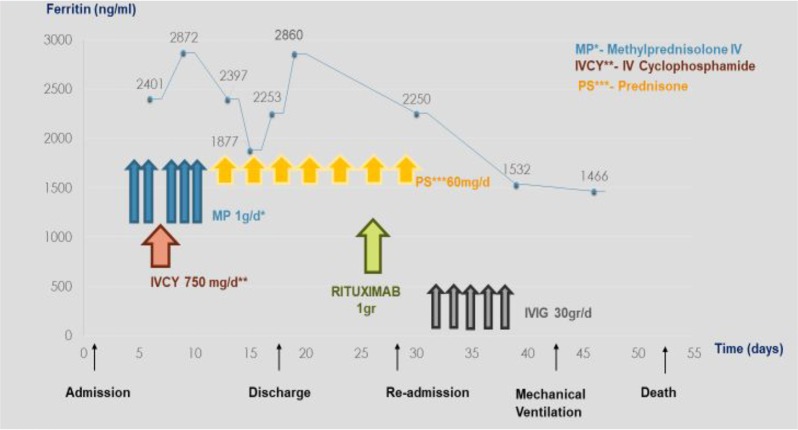
The correlation between disease progression, serum ferritin level and immunomodulatory therapies.

Before his discharge, the serum ferritin level remained high 2860ng/mL (*Figure 2*) and triglycerides level were increased up to 389mg/dl. Two weeks later, he received Rituximab (1mg/d). At that point, the patient felt improvement in his respiratory condition. However, a day later, he was admitted due to fever and chills. In arrival, room air saturation was 89%, the sputum analyzed for PCP and aspergillus were negative. An additional chest CT showed a diffuse lung disease (*Figure 1B*). Therapy with intravenous steroids and immunoglobulins was given for additional five days without any clinical improvement, however, the ferritin level dropped to 1466ng/mL (*Figure 2*). A progressive respiratory failure led to mechanical ventilation and eventually, on day 52 since his first admission, the patient passed away.

## DISCUSSION

Initially anti-MDA5 was called Anti-CADM 140, owing to its identification in sera of Japanese patients with clinically amyopathic dermatomyositis, in particular, those with rapidly progressive interstitial lung disease.^1^ Beyond respiratory features, the clinical presentation of anti-MDA5 positive dermatomyositis is characterized by pathognomonic dermatological lesions including painful palmar erythematous papules and cutaneous ulcers on metacarpophalangeal joints, lateral nail folds, elbows and knees.^2^ Our patient did not exhibit those specific dermatological signs. An important prognostic biomarker is a serum ferritin level which is correlated with disease activity, treatment responsivity and survival outcome.^3^ Pre-treatment ferritin level higher than 1600ng/ml is associated with a more severe disease and a lower survival rate.^3^ The exact pathophysiology of anti-MDA5 positive dermatomyositis is unknown. Nevertheless, the presence of multiple alveolar macrophages as demonstrated in autopsy specimens of Clinically amyopathic dermatomyositis patient together with high blood ferritin and triglycerides levels raise the suspicion of macrophage activation syndrome as part of the pathogenesis of this disease.^3^ Melanoma differentiation associated gene five is an intracellular protein that acts as a viral sensor and initiates an immune cascade leading to cytotoxicity and fragments production which may induce self-response as anti-MDA5 autoantibody formulation.^2,4^ Although there are no formal therapeutic guidelines, combination of corticosteroids, intravenous cyclophosphamide and calcineurin inhibitors are recommended for dermatomyositis with interstitial lung disease and increase survival rate up to 75%.^4^ Other potential therapies including rituximab and IVIG were recently reported as beneficial.^5^ In our case, unfortunately, the patient had already an irreversible diffuse lung damage that eventually led to respiratory failure and death.

In conclusion, anti-MDA5 associated dermatomyositis with a rapidly progressive interstitial lung disease results in high mortality rates. The pathognomonic dermatological features are not always crucial for diagnosis, yet, the combination of respiratory symptoms with the detection of anti-mda5 or anti aminoacyl-tRNA synthetase confirms the diagnosis of dermatomyositis. At this point, expeditious initiation of therapy should be done, before irreversible damage occurs, in order to impede disease progression and affect survival outcomes. In addition, repetitive measurements of serum ferritin level may aid in evaluation of treatment response.
